# A Smartphone Remote Monitoring App to Follow Up Colorectal Cancer Survivors: Requirement Analysis

**DOI:** 10.2196/18083

**Published:** 2022-01-05

**Authors:** Seyed Mohammad Ayyoubzadeh, Mohammad Shirkhoda, Sharareh R Niakan Kalhori, Niloofar Mohammadzadeh, Somayyeh Zakerabasali

**Affiliations:** 1 Department of Health Information Management School of Allied Medical Sciences Tehran University of Medical Sciences Tehran Iran; 2 Department of Surgery Cancer Institute of Iran Tehran University of Medical Sciences Tehran Iran; 3 Peter L. Reichertz Institute for Medical Informatics Technical University Braunschweig and Hannover Medical School Braunschweig Germany; 4 Clinical Education Research Center, Health Human Resources Research Center School of Health Management and Information Sciences Shiraz University of Medical Sciences Shiraz Iran

**Keywords:** eHealth, app, colorectal cancer, survivors, requirements analysis, MoSCoW

## Abstract

**Background:**

Colorectal cancer survivors face multiple challenges after discharge. eHealth may potentially support them by providing tools such as smartphone apps. They have lots of capabilities to exchange information and could be used for remote monitoring of these patients.

**Objective:**

In this study, we addressed the required features for apps designed to follow up colorectal cancer patients based on survivors’ and clinical experts’ views.

**Methods:**

A mixed methods study was conducted. Features of related apps were extracted through the literature; the features were categorized, and then, they were modified. A questionnaire was designed containing the features listed and prioritized based on the MoSCoW (Must have, Should have, Could have, Won’t have) technique and an open question for each category. The link to the questionnaire was shared among clinical experts in Iran. The answers were analyzed using the content validity ratio (CVR), and based on the value of this measure, the minimum feature set of a monitoring app to follow up patients with colorectal cancer was addressed. In addition, a telephone interview with colorectal cancer survivors was conducted to collect their viewpoints regarding a remote monitoring system for colorectal cancer cases.

**Results:**

The questionnaire contained 10 sections evaluating 9 categories of features. The questionnaire was completed by 18 experts. The minimum set of features in the app was identified as patient information registration, sign and symptom monitoring, education, reminders, and patient evaluation (0.42 < CVR < 0.85). Features including physical activity, personalized advice, and social network did not achieve the minimum score (–0.11 < CVR < 0.39). We interviewed 9 colorectal cancer survivors. Information registration, sign and symptom monitoring, education, and personalized advice were the features with high priority from the survivors’ perspectives. Scheduling, shopping, and financial support features were emphasized by survivors in the interview.

**Conclusions:**

The requirement set could be used to design an app for the targeted population or patients affected by other cancers. As the views from both survivors and clinical experts were considered in this study, the remote system may more adequately fulfill the need for follow-up of survivors. This eases the patients’ and health care providers’ communication and interaction.

## Introduction

Colorectal cancer is one of the most prevalent cancers in the world [1]. Nowadays, with the improvement in health care systems, the number of survivors of this cancer has increased [2]. These survivors face multiple challenges, including a high risk of cancer recurrence. In addition, a high percentage may experience comorbidities from treatment [3].

Financial limitations and pressures caused by the services provided to cancer patients in the health care system result in discharge from the hospital earlier [4]. Therefore, postdischarge care of people with chronic illnesses such as cancer is essential to reduce their readmission [5].

eHealth tools provide a great opportunity to decrease the hospital length of stay and improve care for these survivors. In addition, after discharge, these tools can be used for symptom monitoring, physical activity tracking, psychological issues related to cancer, and nutrition management as well as undergoing a consultation from physicians and health care providers [6].

Positive effects of eHealth interventions on cancer patients’ psychological health, appropriate control of their symptoms, communication, knowledge and skills, and quality of life have been reported [4]. The findings have shown cancer survivors’ active engagement in their health management [4]. This has led to a major shift from hospital care to informal care at home [7] and patients’ attitudes toward self-care and self-management [8].

There is some evidence that recent technologies such as web-based programs [9,10] and smartphone apps [11] can meet information needs related to cancer patients’ diagnoses and prognosis management at home. This performance requires that the content and features of these technologies are based on intended users’ needs.

Smartphone technologies are rapidly expanding in the health care system due to their availability and ease of use [12] and have a lot of potential for providing access to information, support, and resources from anywhere [13]. However, a limited number of these smartphone apps is devoted to remote monitoring of chronic cancer, especially colorectal cancer, based on the patients’ situations to support self-care and making the right decision at each stage of treatment. For example, according to a recent study, 63% of these apps were devoted to diabetes, and only 5% of them related to cancer, and then mainly for information delivery [14].

Consequently, smartphone apps can play an effective role in helping with the follow-up of patients with colorectal cancer, remote monitoring of physical and mental signs and symptoms, and improving health care through patient understanding of what they need to do in each phase via an easy electronic consult with their clinical experts. Therefore, the purpose of this study was to identify and analyze the required features of remote monitoring smartphone apps designed to follow up colorectal cancer survivors with the focus of supporting them after surgery.

## Methods

A cross-sectional, mixed methods study was designed to determine the requirements for a smartphone app to monitor colorectal cancer survivors after discharge and was conducted in 2019. The requirements for this smartphone app were gathered from a previous study that investigated eHealth tools for supporting colorectal cancer survivors, by reviewing articles [15-29]. The features of the apps introduced in these articles were extracted and reviewed by a group of medical informatics experts (n=3) and validated by a clinical specialist (n=1, MS). Then, a questionnaire was created containing the requirements based on the extracted features. The questionnaire was generated on an online questionnaire builder platform, and the link to the questionnaire was shared via Telegram messenger in a group including oncological surgeons and related clinical experts.

The questionnaire was composed of 10 parts. Except for the first part that was designed to gather the responders’ information, the next 9 parts were designed to obtain the experts` opinions about apps requirements (or the so-called features). The questionnaire structure is shown in Table 1.

For sections 2 to 8 of the questionnaire, items were scaled as 4-choice questions based on the MoSCoW (Must have, Should have, Could have, Won’t have) method [30], and at the end of each section, an open question regarding comments on that section was asked.

MoSCoW is a technique used for requirement prioritization. This technique categorizes each requirement into “Must have,” “Should have,” “Could have,” and “Won’t have” requirements. The “Must-have” requirements indicate that the feature must be implemented in this version. The “Should have” requirements indicate that the features must be implemented in this version if at all possible. The “Could have” requirements indicate that the features could be implemented if they do not affect any other requirement. The “Won’t have” requirements indicate features that are not needed in this version but could be included in the future.

In the next phase, the content validity ratio (CVR) was determined using the formula:



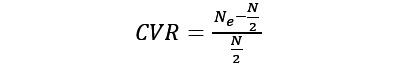



where N is the total number of experts (n=18) and *N_e_* refers to the count of experts that chose “Must have” or “Should have” to consider the features as essential requirements. The threshold was considered based on the nearest original thresholds introduced by Lawshe [31]. In his work, he provided a table containing the minimum required CVR for an item to be selected based on the number of content evaluation panel members. In this study, the threshold was set at 0.42, based on the table by Lawshe [31].

**Table 1:** Questionnaire items designed to obtain the experts’ opinions about the required features of apps designed to follow up colorectal cancer survivors.

Section codes, section names, and item codes		Items
**Section 0: Questionnaire responder information**		
	Item 0.1	Name (optional)
	Item 0.2	Gender
	Item 0.3	Expertise
	Item 0.4	Work experience duration
	Item 0.5	Activity type
	Item 0.6	City
	Item 0.7	Cell number (optional)
	Item 0.8	email address (optional)
**Section 1: Patient information registration**		
	Item 1.1	Sociodemographic information
	Item 1.2	Diagnosis and previous surgery information
	Item 1.3	Surgery and after surgery information
	Item 1.4	Comments on this section
**Section 2: Sign and symptom monitoring**		
	Item 2.1	Weight tracking
	Item 2.2	Vital sign tracking
	Item 2.3	Symptom tracking
	Item 2.4	Side effect tracking
	Item 2.5	BMI tracking
	Item 2.6	Comments on this section
**Section 3: Education**		
	Item 3.1	Information about cancer
	Item 3.2	Common issues for patients
	Item 3.3	Information about physical activity
	Item 3.4	Information about drugs
	Item 3.5	Information about chemotherapy
	Item 3.6	Information about nutrition
	Item 3.7	Information about rehabilitation
	Item 3.8	Information about the treatment process
	Item 3.9	Information about postdischarge
	Item 3.10	Information about pain management
	Item 3.11	Information about emergency issue management
	Item 3.12	Other patients' experiences
	Item 3.13	Comments on this section
**Section 4: Physical activity**		
	Item 4.1	Goal setting for physical activity
	Item 4.2	Physical activity tracking
	Item 4.3	Viewing other patients’ physical activity progress
	Item 4.4	Comments on this section
**Section 5: Reminders**		
	Item 5.1	Hospital visit reminders
	Item 5.2	Medication reminders
	Item 5.3	Comments on this section
**Section 6: Personalized advice**		
	Item 6.1	Online consultation system
	Item 6.2	Tailored patient information
	Item 6.3	Comments on this section
**Section 7: Patient evaluation**		
	Item 7.1	Quality of life evaluation
	Item 7.2	Nutrition evaluation
	Item 7.3	Clinician-patient relationship evaluation
	Item 7.4	Comments on this section
**Section 8: Social network**		
	Item 8.1	Patients’ discussion groups
	Item 8.2	Comments on this section
**Section 9: Comments**		
	Item 9.1	Other comments

In the second phase, a one-on-one, phone, semistructured interview was conducted to ask colorectal cancer survivors’ opinions about a mobile app’s features. The open question and the guide questions were designed and finalized based on the research team’s (SMA, SRNK) ideas. The list of colorectal cancer survivors who had already visited the Cancer Institute of Imam Khomeini hospital in Tehran and were discharged during the last year (2019-2020) was prepared, and sampling was conducted randomly. The telephone numbers of all the patients were available, and the researcher called them to ask the designed questions. Unfortunately, 5 calls resulted in the very bad news of the patient’s death after discharge. The interview was conducted with the remaining 9 survivors or one of their family members.

The guide for asking the open question was “What features do you think a mobile app needs to help you for better care? Or do you have any health problems that an app could help you in that condition?”

After asking the open question, the survivors were asked to prioritize the feature categories, by scoring each feature category from 1 (the lowest priority) to 4 (highest priority).

The interviews were transcribed, coded, and categorized into themes for qualitative analysis. In the quantitative analysis, to rank the features based on scores, the CVR of each category was calculated, and the threshold was set at 0.78 based on the thresholds by Lawshe (n=9).

## Results

### Clinical Experts’ Information

The questionnaire was completed by 18 experts (3 women, 15 men): 7 were oncological surgeons, 5 were general surgeons, and 3 were clinical oncologists. The others included an internist, a laparoscopic specialist, and one who did not specify his expertise. Most (11/18, 61%) possessed a work experience of 5 years to 10 years. They were working mostly in Tehran, the capital of Iran. Others were working in the other 7 cities in the country. One worked in the 2 cities of Tehran and Khorasan concurrently. The responders’ characteristics are shown in Table 2.

**Table 2:** Responders’ characteristics (n=18).

Characteristics		Value, n (%)
**Gender**		
	Female	3 (17)
	Male	15 (83)
**Specialty**		
	General surgeon	5 (28)
	Oncology surgeon fellowship	7 (39)
	Clinical oncologist	3 (17)
	Other	3 (17)
**Work experience**		
	Less than 5 years	3 (17)
	Between 5 and 10 years	11 (61)
	Between 11 to 15 years	1 (6)
	Between 16 to 20 years	1 (6)
	More than 20 years	2 (11)
**Faculty**		
	Faculty member	11 (61)
	Not faculty member	7 (39)
**City**		
	Tehran	7 (39)
	Tehran and Khorasan	1 (6)
	Yazd	2 (11)
	Mashhad	1 (6)
	Gorgan	1 (6)
	Gerash	1 (6)
	Shiraz	1 (6)
	Sanandaj	1 (6)
	Dezful	1 (6)
	Ahvaz	1 (6)
	Isfahan	1 (6)

### Requirement Prioritization

The responses to the 4-choice questions based on the MoSCoW method and the CVR for each item are represented in Table 3. All experts had consensus on the “Diagnosis and previous surgery information” and “Surgery and after surgery information” items.

Based on CVR values for each item shown in Table 3 and the threshold (0.42), 21 items should be considered as essential requirements, categorized in 8 main groups.

**Table 3:** Responses to the 4-choice questions and content validity ratio (CVR) for each item (n=18).

Item			Must have, n		Should have, n		Could have, n		Won’t have, n		CVR
**Patient information registration (overall CVR=0.85)**											
	Sociodemographic information	10		4		4		0		0.56	
	Diagnosis and previous surgery information	18		0		0		0		1.00	
	Surgery and after surgery information	18		0		0		0		1.00	
**Sign and symptoms monitoring (overall CVR=0.42)**											
	Weight tracking	9		6		3		0		0.67	
	Vital sign tracking	5		4		7		2		0.00	
	Symptom tracking	6		4		7		1		0.11	
	Side effect tracking	16		2		0		0		1.00	
	BMI tracking	5		7		5		1		0.33	
**Education (overall CVR=0.58)**											
	Information about cancer	10		3		4		1		0.44	
	Common issues for patients	13		2		3		0		0.67	
	Information about physical activity	6		6		5		1		0.33	
	Information about drugs	8		5		3		2		0.44	
	Information about chemotherapy	9		7		2		0		0.78	
	Information about nutrition	14		3		1		0		0.89	
	Information about rehabilitation	12		5		1		0		0.89	
	Information about treatment process	12		1		4		1		0.44	
	Information about post discharge	13		4		1		0		0.89	
	Information about pain management	9		5		3		1		0.56	
	Information about emergency issue management	14		3		1		0		0.89	
	Other patients experience	5		2		8		3		–0.22	
**Physical activity (overall CVR=–0.22)**											
	Goal setting for physical activity	3		4		9		2		–0.22	
	Physical activity tracking	4		4		8		2		–0.11	
	Viewing other patients’ physical activity progress	4		2		9		3		–0.33	
**Reminders (overall CVR=0.84)**											
	Hospital visit reminder	14		3		1		0		0.89	
	Medication reminder	12		4		2		0		0.78	
**Personalized advice (overall CVR=0.39)**											
	Online consultation system	3		9		5		1		0.33	
	Tailored patient information	7		6		5		0		0.44	
**Patient evaluation (overall CVR=0.70)**											
	Quality of life evaluation	10		5		3		0		0.67	
	Nutrition evaluation	12		6		0		0		1.00	
	Clinician-patient relationship evaluation	9		4		5		0		0.44	
**Social network (overall CVR=**–**0.11)**											
	Patients’ discussion groups	1		7		8		2		–0.11	

### Open Question Responses

One specialist commented on section one and stated that “the patient’s history with rich data could be helpful for retrospective studies and will increase the importance of the app.”

Two other specialists commented on the items of section three. One asked the following question: “To which extent the data will be provided to the patient?” In addition, the other had concerns about sharing other patient experiences by commenting: “the patients’ experiences are usually not scientific and have no profound evidence.” This expert also commented on the items in section eight, by stating “I disagree with all kinds of opinion exchange with patients. Because unfortunately, most of the time it led to wrong information transfer.” Another expert also commented on this section by stating “regular group sessions with clinicians and patients [will be helpful].”

In the last section, 3 experts left their comments. One noted that: “I believe that [an app with these features] are too useful.” The other hoped success for the research team. The third addressed 3 issues:

(1) regarding patients with colostomy, the app should provide exact information about the bag and notes for changing that; (2) possible emergency after surgery such as fever and infection, gastrointestinal hemorrhage, thromboembolic, and signs and symptoms of recurrence should be informed to the patient; (3) patients have interest in [care] details such as nutrition, physical activity, etc.; in this app, these issues should be noticed.

### Telephone Interviews With Colorectal Cancer Survivors

The survivors’ characteristics are shown in Table 4.

The themes extracted from the interviews were accessibility and usability; 2-sided information flow, to inform and get informed; scheduling; shopping; decision-making; social support; and financial support.

**Table 4:** Colorectal cancer survivors’ characteristics (n=9).

Characteristics		Value, n (%)
**Gender**		
	Female	5 (56)
	Male	4 (44)
**Age (years)**		
	50-59	3 (33)
	60-69	4 (44)
	70-79	2 (22)
**City**		
	Tehran	3 (33)
	Arak	1 (11)
	Sanandaj	1 (11)
	Karaj	1 (11)
	Elam	1 (11)
	Borujerd	1 (11)
	Khoramabad	1 (11)
**Cancer**		
	Colon	8 (89)
	Rectum	1 (11)
**Chemotherapy**		
	Yes	8 (89)
	No	1 (11)

#### Accessibility and Usability

One survivor mentioned she does not have a smartphone. Another mentioned an older adult survivor could not work with the app, although a member of the family may help and work with the system instead.

#### 2-Sided Information Flow, to Inform and Get Informed

The survivors wanted to be monitored. The app should ask questions about the survivor’s health and inform the clinician about the survivor’s status. The survivors could ask questions regarding what to do with nutrition, losing weight, high blood pressure, and other challenges. The survivors wanted to know what to do in each condition. They need a consultation. They become anxious about unknown side effects. The app should provide information about colostomy bags. In addition, the possibility of sending high-quality lab results to the clinician via an electronic tool was requested.

#### Scheduling

The survivors complained of the scheduling process, and they requested that the app have features to ease the scheduling process, especially for those who are traveling from cities located far across the country from Tehran where more highly specialized physicians are available.

#### Shopping

Some survivors complained of the hardness to get and find medications for their cancer and asked for a feature in the app to sell medications and colostomy bags.

#### Decision-making

One survivor pointed out that the app should log medication intake and the outcomes of taking each medication. This would enable clinicians to make better decisions.

#### Social Support

One survivor mentioned that the discussion group can provide social support.

#### Financial Support

Some patients were deeply unhappy about the cost of a colostomy bag, traveling, medication, and even access to smartphone expenses. The app might help them to be connected with donations and charity organizations.

The survivors acknowledged the app helps the survivors, especially those who are in cities other than their clinician’s city (in this case, Tehran) because of traveling. Furthermore, they pointed out that such an app is more useful in the COVID-19 pandemic.

The priority of feature categories is shown in Table 5.

**Table 5:** Responses to the 4-choice questions (n=9).

Item	Highest priority (4), n	Above medium priority (3), n	Medium priority (2), n	Lowest priority (1), n	CVR^a^
Patient information registration	7	1	1	0	0.78
Sign and symptom monitoring	7	2	0	0	1.00
Education	9	0	0	0	1.00
Physical activity	4	3	1	1	0.56
Reminders	5	2	2	0	0.56
Personalized advice	6	2	1	0	0.78
Patient evaluation	3	4	1	1	0.56
Social network	5	1	1	2	0.33

^a^CVR: content validity ratio.

## Discussion

### Principal Findings

In this study, the requirements for a remote monitoring smartphone app to follow up colorectal cancer survivors were extracted and prioritized using the MoSCoW method and CVR. The proposed questionnaire was completed by 18 specialists, and an interview was conducted with 9 colorectal cancer survivors.

Although a successful app should consider all stakeholders in the design process [32], the patients, as one of the primary stakeholders, should be involved in the design. Thus, it is necessary to listen to and get feedback from patients during the elaboration of the design and prototyping the app. In this study, we addressed the specialists’ view of such an app. The findings align with those of previous studies that addressed colorectal cancer survivors’ needs, which we discuss in the following paragraphs.

Information about the diagnosis and treatment summary as well as medical and nonmedical needs were shown to be useful for colorectal cancer survivors. Other needs, such as information about late effects and likely issues including fatigue and bowel-related symptoms, could be helpful for them. They also need information about nutrition and general health. A list of recommended tests is also important for survivors. In addition, information about cancer, prognosis, and recurrence of cancer are suitable. Some survivors may prefer to be informed about finding a local health care center for ongoing care, personalized information, and trusted sources of information [33]. Generally, the met and unmet needs of colorectal cancer survivors could be categorized as physical symptoms, emotional, information, and coping strategies [34]. The designed app should address these information needs of the colorectal cancer survivors. However, previous apps designed for cancer survivors have not satisfied all the information needs of these survivors such as psychological support, managing finances, and long-term effects [35]. A comprehensive app addressing these needs, as included in the requirements analysis, might be more supportive for survivors.

The 2 items entitled “Diagnosis and previous surgery information” and “Surgery and after surgery information” were added to the questionnaire based on the expert author’s (MS) comment. These 2 items had a CVR of 1, which means all experts had consensus on these items. In addition, from the survivors’ view, this feature was necessary. “Sign and symptom monitoring” was extremely important from the survivors’ point of view, and, similarly, “Side effect tracking” also had a CVR of 1, which might be due to the high rate of colorectal cancer comorbidities and treatment effects, as mentioned before in [3] in which 18% of patients with colorectal cancer experienced at least one comorbidity after their discharge.

The “reminders” domain, including items for hospital visit reminders and medication reminders, had a high score, indicating the experts believed that reminders are important and the app could manage them effectively. In contrast, reminders were not considered essential from the survivors’ point of view, although most of them thought reminders were of high priority. One underlying reason may be reflected by one survivor’s view that “families are engaged in the health care, and there is no need for reminders.”

The education domain, except physical activity training, and the reminder domain could be effectively implemented from the experts’ point of view. This domain is considered essential from the survivors’ point of view.

Physical activity has been shown to be effective in reducing colorectal cancer mortality [36] and its negative effects [37]. However, the “physical activity” domain in addition to the “Information about physical activity” items in this study did not reach the minimum score to be in the minimum feature set of such a smartphone app. The reason may be related to studies such as [15,24] that showed eHealth is not effective in improving physical activity behaviors of cancer patients. Some survivors mentioned that they could not perform physical activity due to their condition. Perhaps, for younger survivors, this feature would be marked as essential.

The “patients’ discussion groups” domain did not pass the test; this might be represented by the statement of one expert: “the patient’s experiences are usually not scientific and have no profound evidence.” Thus, this causes the problem of misinformation exchange between patients. Some patients had the same idea about this feature. However, other patients mentioned that it could be useful to know the trajectory of other patients and that it could provide social support.

The “personalized advice” domain was a priority for survivors, although the clinicians did not consider consultations as a high-priority feature. The reason may be the lack of clinicians’ time and that the costs are not covered by any party.

As mentioned by one of the experts, training and notes about the use of a colostomy bag should be considered in the app, particularly in the education section. Regarding the other comment from an expert who stated the app could be helpful, it is worth mentioning that his or her comment could be true in general. However, the effectiveness of these apps in the domains of survivors’ nutritional status and social support has not been shown [38].

**Figure 1:**
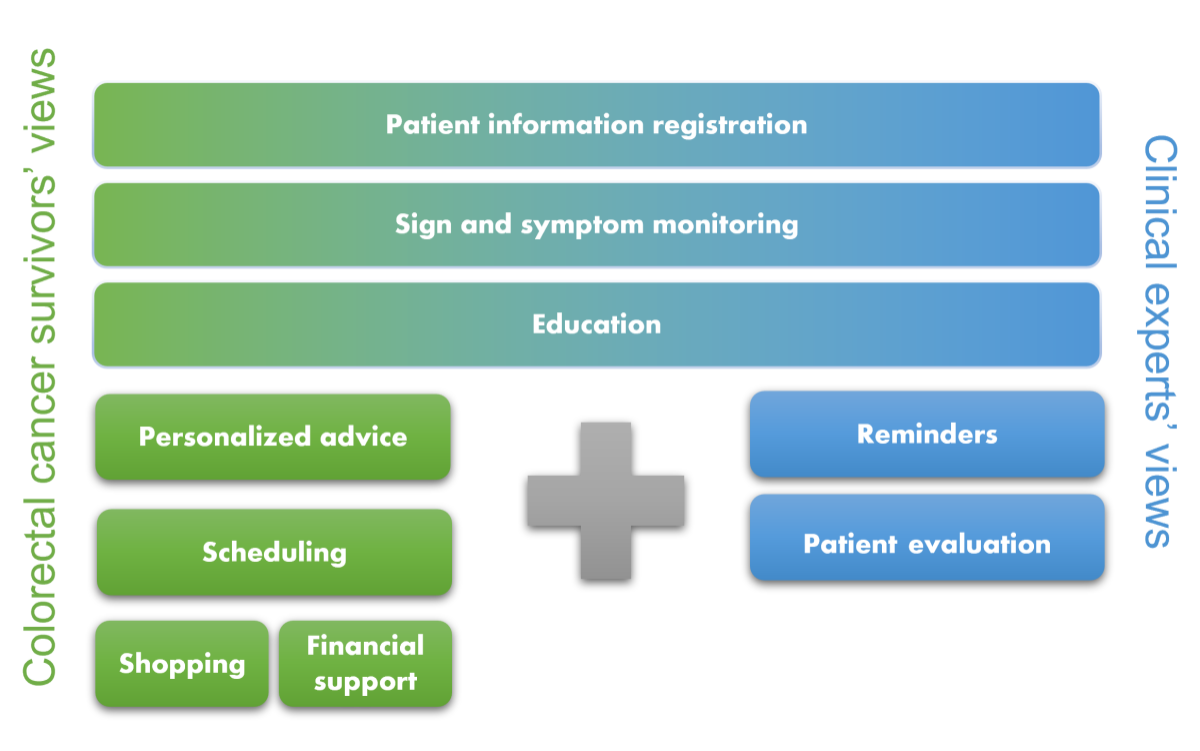
Requirements for a smartphone app to remotely monitor and follow up colorectal cancer survivors.

All the high-priority requirements are shown in Figure 1. The requirements were gathered form the combination of the colorectal cancer survivors’ and clinical experts’ priorities based on the CVR and items mentioned by more than one survivor in the interviews.

Overall, the findings suggest that a smartphone app to remotely monitor patients with colorectal cancer after discharge should be designed to include the features of patient information registration, sign and symptom monitoring, education, reminders, patient evaluation, personalized advice, scheduling, shopping, and financial support.

Recently, especially during the COVID-19 outbreak, eHealth interventions have gained more attention from governments and populations. They are showing more encouragement for remote interventions. This could lead to progress in investment in such apps. The suitable design and standardization of features for these apps may help to better provide support to these survivors. In this study, the features of the app were defined based on experts’ views; future works could focus on obtaining patients’ views and elaborating these features.

### Strengths and Limitations

One of the strengths of this study was the provision of a feature set for a remote monitoring and educational app for colorectal cancer survivors after surgery for the first time in the country. In addition, the combination of qualitative questions and quantitative methods and obtaining clinical experts’ and survivors’ viewpoints is an approach that could also be considered a strength. Another strength is the collaboration between oncological experts and the research team.

The research was conducted in Iran, and the priorities for the features could be different in other countries. Thus, this could be considered a limitation of this study. The sample size could also be considered a limitation.

### Conclusion

In this study, the requirements of a remote monitoring smartphone app to follow up colorectal patients were determined by literature review, specialists’ confirmation, and survivors’ viewpoints. These requirements might help design such an app. Further research should address the generalizability of the feature set in other cancers and the possibility of defining standards for such apps.

## Abbreviations

CVR: content validity ratio
MoSCoW: Must have, Should have, Could have, Won’t have

## References

[1] (2018). Cancer. World Health Organization.

[2] Allemani C, Matsuda T, Di Carlo V, Harewood R, Matz M, Nikšić M, Bonaventure A, Valkov M, Johnson CJ, Estève J, Ogunbiyi OJ, Azevedo E Silva G, Chen WQ, Eser S, Engholm G, Stiller CA, Monnereau A, Woods RR, Visser O, Lim GH, Aitken J, Weir HK, Coleman MP, CONCORD Working Group (2018). Global surveillance of trends in cancer survival 2000-14 (CONCORD-3): analysis of individual records for 37 513 025 patients diagnosed with one of 18 cancers from 322 population-based registries in 71 countries. Lancet.

[3] Denlinger CS, Engstrom PF (2011). Colorectal cancer survivorship: movement matters. Cancer Prev Res (Phila).

[4] Heynsbergh N, Heckel L, Botti M, Livingston PM (2018). Feasibility, useability and acceptability of technology-based interventions for informal cancer carers: a systematic review. BMC Cancer.

[5] Heynsbergh N, Botti M, Heckel L, Livingston PM (2019). Caring for the person with cancer and the role of digital technology in supporting carers. Support Care Cancer.

[6] Schinköthe T (2019). Individualized eHealth support for oncological therapy management. Breast Care (Basel).

[7] Heynsbergh N, Botti M, Heckel L, Livingston PM (2018). Caring for the person with cancer: Information and support needs and the role of technology. Psychooncology.

[8] Salimian N, Ehteshami A, Ashouri-Talouki M (2019). Developing Ghasedak: a mobile application to improve the quality of cancer palliative care. Acta Inform Med.

[9] Badr H, Carmack CL, Diefenbach MA (2015). Psychosocial interventions for patients and caregivers in the age of new communication technologies: opportunities and challenges in cancer care. J Health Commun.

[10] Kaltenbaugh D, Klem ML, Hu L, Haines A, Hagerty Lingler J, Turi (2015). Using Web-based interventions to support caregivers of patients with cancer: a systematic review. Oncol Nurs Forum.

[11] Bender JL, Yue RYK, To MJ, Deacken L, Jadad AR (2013). A lot of action, but not in the right direction: systematic review and content analysis of smartphone applications for the prevention, detection, and management of cancer. J Med Internet Res.

[12] Saghaeiannejad-Isfahani S, Ehteshami A, Savari E, Samimi A (2017). Developing the medication reminder mobile application "Seeb". Acta Inform Med.

[13] Heynsbergh N, Heckel L, Botti M, Livingston PM (2019). A smartphone app to support carers of people living with cancer: a feasibility and usability study. JMIR Cancer.

[14] Andebe N, Wagacha P, Weru J (2017). Weru, mHealth in palliative care for cancer patients & caregivers.

[15] Mayer DK, Landucci G, Awoyinka L, Atwood AK, Carmack CL, Demark-Wahnefried W, McTavish F, Gustafson DH (2018). SurvivorCHESS to increase physical activity in colon cancer survivors: can we get them moving?. J Cancer Surviv.

[16] Maxwell-Smith C, Cohen PA, Platell C, Tan P, Levitt M, Salama P, Makin GB, Tan J, Salfinger S, Kader Ali Mohan GR, Kane RT, Hince D, Jiménez-Castuera R, Hardcastle SJ (2018). Wearable Activity Technology And Action-Planning (WATAAP) to promote physical activity in cancer survivors: Randomised controlled trial protocol. Int J Clin Health Psychol.

[17] Kim B, Park K, Ryoo S (2018). Effects of a Mobile Educational Program for Colorectal Cancer Patients Undergoing the Enhanced Recovery After Surgery. Open Nurs J.

[18] Golsteijn RHJ, Bolman C, Volders E, Peels DA, de Vries H, Lechner L (2018). Short-term efficacy of a computer-tailored physical activity intervention for prostate and colorectal cancer patients and survivors: a randomized controlled trial. Int J Behav Nutr Phys Act.

[19] Cheong IY, An SY, Cha WC, Rha MY, Kim ST, Chang DK, Hwang JH (2018). Efficacy of mobile health care application and wearable device in improvement of physical performance in colorectal cancer patients undergoing chemotherapy. Clin Colorectal Cancer.

[20] van der Hout A, van Uden-Kraan CF, Witte BI, Coupé VMH, Jansen F, Leemans CR, Cuijpers P, van de Poll-Franse LV, Verdonck-de Leeuw IM (2017). Efficacy, cost-utility and reach of an eHealth self-management application 'Oncokompas' that helps cancer survivors to obtain optimal supportive care: study protocol for a randomised controlled trial. Trials.

[21] Van Blarigan E, Van Loon K, Kenfield SA, Chan JM, Fukuoka Y, Laffan A, Mitchell E, Chan H, Rodriguez-Jaquez D, Meyerhardt JA, Venook AP (2017). Self-monitoring and reminder texts to increase physical activity after colorectal cancer (Smart Pace): A pilot trial. JCO.

[22] Maguire R, Fox PA, McCann L, Miaskowski C, Kotronoulas G, Miller M, Furlong E, Ream E, Armes J, Patiraki E, Gaiger A, Berg GV, Flowerday A, Donnan P, McCrone P, Apostolidis K, Harris J, Katsaragakis S, Buick AR, Kearney N (2017). The eSMART study protocol: a randomised controlled trial to evaluate electronic symptom management using the advanced symptom management system (ASyMS) remote technology for patients with cancer. BMJ Open.

[23] Giesler JM, Keller B, Repke T, Leonhart R, Weis J, Muckelbauer R, Rieckmann N, Müller-Nordhorn J, Lucius-Hoene G, Holmberg C (2017). Effect of a website that presents patients' experiences on self-efficacy and patient competence of colorectal cancer patients: web-based randomized controlled trial. J Med Internet Res.

[24] Forbes CC, Blanchard CM, Mummery WK, Courneya KS (2017). A pilot study on the motivational effects of an internet-delivered physical activity behaviour change programme in Nova Scotian cancer survivors. Psychol Health.

[25] Bray VJ, Dhillon HM, Bell ML, Kabourakis M, Fiero MH, Yip D, Boyle F, Price MA, Vardy JL (2017). Evaluation of a Web-Based Cognitive Rehabilitation Program in Cancer Survivors Reporting Cognitive Symptoms After Chemotherapy. J Clin Oncol.

[26] Drott J, Vilhelmsson M, Kjellgren K, Berterö C (2016). Experiences with a self-reported mobile phone-based system among patients with colorectal cancer: a qualitative study. JMIR Mhealth Uhealth.

[27] Somers TJ, Abernethy AP, Edmond SN, Kelleher SA, Wren AA, Samsa GP, Keefe FJ (2015). A Pilot study of a mobile health pain coping skills training protocol for patients with persistent cancer pain. J Pain Symptom Manage.

[28] Northouse L, Schafenacker A, Barr KLC, Katapodi M, Yoon H, Brittain K, Song L, Ronis DL, An L (2014). A tailored Web-based psychoeducational intervention for cancer patients and their family caregivers. Cancer Nurs.

[29] Schultz PN, Stava C, Beck ML, Vassilopoulou-Sellin R (2003). Internet message board use by patients with cancer and their families. Clin J Oncol Nurs.

[30] Wiegers K, Beatty J (2013). Software requirements.

[31] Lawshe CH (1975). A quantitative approach to content validity. Personnel Psychology.

[32] Abras C, Maloney-Krichmar D, Preece J, Bainbridge W (2004). User-centered design. Encyclopedia of Human-Computer Interaction.

[33] Salz T, Baxi SS, Blinder VS, Elkin EB, Kemeny MM, McCabe MS, Moskowitz CS, Onstad EE, Saltz LB, Temple LK, Oeffinger KC (2014). Colorectal cancer survivors' needs and preferences for survivorship information. J Oncol Pract.

[34] Sutton PA, Bourdon-Pierre R, Smith C, Appleton N, Lightfoot T, Gabriel C, Richards B, Mohamed S, Mason-Whitehead E, Hulbert-Williams NJ, Vimalachandran D (2019). Evaluating unmet needs in patients undergoing surgery for colorectal cancer: a patient reported outcome measures study. Colorectal Dis.

[35] Richards R, Kinnersley P, Brain K, McCutchan G, Staffurth J, Wood F (2018). Use of mobile devices to help cancer patients meet their information needs in non-inpatient settings: systematic review. JMIR Mhealth Uhealth.

[36] Wu W, Guo F, Ye J, Li Y, Shi D, Fang D, Guo J, Li L (2016). Pre- and post-diagnosis physical activity is associated with survival benefits of colorectal cancer patients: a systematic review and meta-analysis. Oncotarget.

[37] Oruç Z, Kaplan MA (2019). Effect of exercise on colorectal cancer prevention and treatment. World J Gastrointest Oncol.

[38] Ayyoubzadeh SM, R Niakan Kalhori S, Shirkhoda M, Mohammadzadeh N, Esmaeili M (2020). Supporting colorectal cancer survivors using eHealth: a systematic review and framework suggestion. Support Care Cancer.

